# A Massive Extradural Hematoma in Sickle Cell Disease and the Importance of Rapid Neuroimaging

**DOI:** 10.1155/2019/1742472

**Published:** 2019-12-18

**Authors:** Per Ole Iversen, Mboka Jacob, Jamila Makame, Mclean Abisay, Mbonea Yonazi, Anna Schuh, Julie Makani

**Affiliations:** ^1^Department of Haematology and Blood Transfusion, Muhimbili University of Health and Allied Sciences, Dar-es-Salaam, Tanzania; ^2^Department of Radiology and Imaging, Muhimbili University of Health and Allied Sciences, Dar-es-Salaam, Tanzania; ^3^Department of Haematology, Muhimbili National Hospital, Dar-es-Salaam, Tanzania

## Abstract

Sickle cell disease (SCD) is an inherited hemoglobinopathy leading to several serious organ complications and early death. It is mostly found in equatorial countries like Tanzania. Extradural hematoma (EDH) is a rare, but serious complication to SCD and may have debilitating consequences. Hitherto, there is no report of EDH in SCD where neuroimaging has been available before, during, and after such an event. Here, we describe a young female SCD patient who developed EDH that required surgical evacuation. She had made full recovery after three months. Neuroimaging performed two years prior to this event was unremarkable except for multiple small cerebral infarcts. On admission, neuroimaging revealed a subgaleal hematoma, possibly indicating disruption of the skull cortex due to increased hematopoiesis. Three months after evacuation of the hematoma, neuroimaging showed evidence of brain atrophy and the previously reported cerebral infarcts and multifocal bone infarction, but no vasculopathy. Possibly, disruption of the skull cortex with subsequent bleeding caused the EDH. As the differential diagnoses of neurological complications in SCD are many and some complications are reversible, neuroimaging should be performed without delay.

## 1. Introduction

Sickle cell disease (SCD) is an autosomal recessive disorder resulting in production of abnormal erythrocytes due to mutation in the gene coding for the beta chain in the hemoglobin (Hb) molecule. SCD is characterized by a range of complications, in particular, anemia, infections, and painful vasoocclusive crisis (VOC) [1]. In addition, silent cerebral infarcts are common in SCD and constitute a risk factor for future cerebral infarcts leading to clinical manifestations [2]. SCD is particularly frequent in Africa and Tanzania which are among the countries with the highest burden of this disease [3].

A severe form of VOC in SCD is extradural hematoma (EDH). Neurological sequelae, impaired cognition, and increased mortality have been reported in SCD patients affected by this complication [4, 5]. The underlying mechanism leading to EDH is not known, but it has been associated with a number of other diseases (e.g., infection, cancer, bleeding disorder, and vasculopathy), as reviewed by Hettige et al. [6]; however, none of them has been convincingly shown to cause EDH. Page et al. suggested that a preexistent bone infarction in SCD could lead to rupture of nearby blood vessels [4]. Alternatively, abnormal skull anatomy in SCD due to increased hematopoiesis with subsequent rupture of the skull cortex could lead to bleeding into the subgaleal and epidural spaces [4, 7]. However, without available neuroimaging data obtained prior to the development of EDH, its cause is difficult to identify.

Due to the rarity, our knowledge of EDH stems from case reports, and in all of them, neuroimaging prior to the bleeding is not available, precluding further examination into the possible causes of this often debilitating SCD complication. To the best of our knowledge, this is the first presentation of a SCD patient suffering from an apparent spontaneous form of massive EDH of whom neuroimaging was available prior to and during this event as well as after rehabilitation.

## 2. Case Presentation

An 18-year-old female from Tanzania was diagnosed with homozygous (HbSS) SCD at one year of age. During childhood and adolescence, she had multiple admissions for painful episodes of bone VOC and infections, but no reported clinical features of affections of the central nervous system. Her steady-state Hb ranged from ∼7 to 8 g/dl; HbS from ∼70 to 80%, and HbF from ∼2 to 11%. She used folic acid (5 mg/day) and intermittently (due to financial constraints) hydroxyurea (0.5–1 g/day).

**Figure 1:**
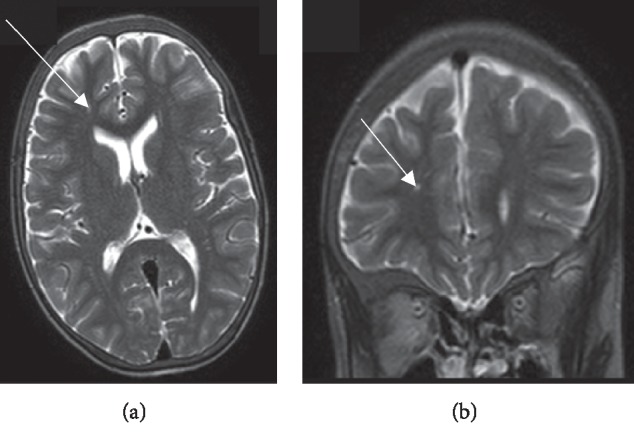
(a) Axial T2-weighted MRI brain scan demonstrating focal hyperintensity (arrow), probably representing an infarctive lesion on the right frontal lobe deep white matter. (b) The same lesion as seen on the coronal T2-weighted MRI brain scan.

Two years prior to the current admission, a brain magnetic resonance imaging (MRI) angiography was performed as a screening for cerebral infarctions and vasculopathy as part of a research programme. This showed multiple small cerebral infarcts, and the largest (∼3 mm) was located in the right frontal deep white matter (Figures 1(a) and 1(b)). There were no features of skull abnormalities or arteriovenous malformations, and all intracranial arteries were unremarkable.

She was first seen at a local hospital before being transported to Muhimbili National Hospital after one day with loss of consciousness. This was preceded by frontal headache three days prior to hospital admission which increased in severity with time. There was no history of trauma, vomiting, convulsions, or urine/fecal incontinence. One week prior to hospital admission, she had bone pain on and off and low-grade fever, which were relieved by analgesia and antipyretics, respectively.

On arrival at Muhimbili National Hospital, the patient was unconscious with a Glasgow Coma Scale score of 7/15 and febrile (38.9°C), with moderate pallor, but no jaundice. The pupils were dilated and sluggishly reacting to light. In addition, she had developed right-sided hemiparesis. There was no visible evidence of head trauma or signs of meningeal affection.

**Figure 2:**
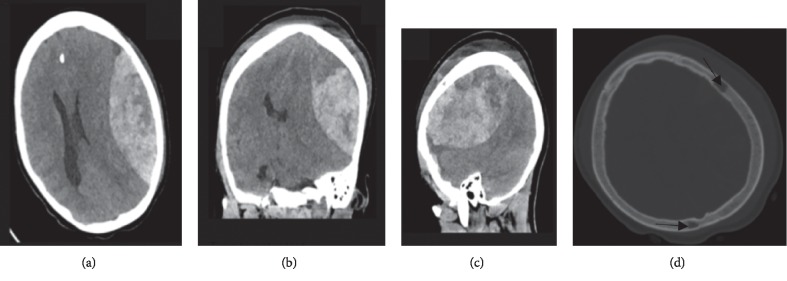
(a) Axial CT brain scan (no contrast) demonstrating left-sided extradural hematoma. (b and c) Coronal and sagittal CT brain scans, respectively, showing the extradural hematoma and subgaleal hemorrhage. (d) The preoperative CT on bone window showed two hypodense lesions (arrows) on the left frontal and right parietal bones.

A computerized tomography (CT) scan of the brain on admission revealed a massive biconvex extradural heterogeneous (predominantly hyperdense) hematoma (4.37 cm in maximum thickness with 1.23 cm of midline shift) overlying the left frontal and parietal lobes (Figures 2(a) and 2(b)). The hematoma compressed the underlying brain matter so that the gyral and sulci pattern was lost and hypodense indicating edema. Moreover, an effaced ipsilateral lateral ventricle and a moderate dilatation of the contralateral lateral ventricle had developed. A subgaleal hematoma overlying the left frontal and parietal bones was also noted (Figures 2(b) and 2(c)). There was no skull lesion or features or signs of osteomyelitis. In addition, we noted two hypodense lesions consistent with bone infarction on the left frontal and right parietal bones (Figure 2(d)).

Her Hb was 5.0 (reference value: 12.0–15.0) g/dl, leukocytes 6.1 (4.0–10.0) × 10^9^/l, and platelets 73.0 (150–410) × 10^9^/l. The concentration of C-reactive protein was elevated (287; 0–5) mg/l, while screenings for malaria, urinary tract, typhoid, and dengue infections were all negative. Additional blood tests revealed no hypercoagulability or bleeding tendency, including normal INR and APTT.

**Figure 3:**
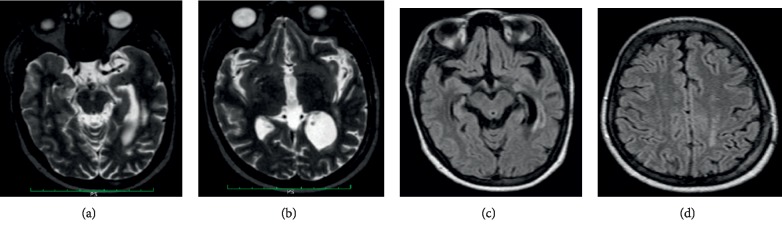
MRI brain scan performed three months after evacuation of the extradural hematoma. (a) Axial T2-weighted MRI scan showing multifocal hyperintensities on the left periventricular area and dilated posterior horn of the left lateral ventricle. (b) Coronal T2-weighted MRI scan and (c) axial “fluid attenuated inversion recovery (FLAIR)-MRI. FLAIR-MRI are used to enhance T2-weighted scans to identify hyperintense lesions by suppressing cerebrospinal fluid. Both images (b and c) show prominent left sylvian fissure and dilated posterior horn of the left lateral ventricle and left temporal lobe atrophy. (d) Axial FLAIR-MRI showing white matter hyperintensity of the left parietal lobe signal changes on the parietal bone.

Due to progressive deterioration of her condition, the patient underwent a left parietal craniotomy and evacuation of the EDH under general anesthesia. She regained consciousness the day after surgery; however, she remained aphasic with right-sided hemiparesis for 3-4 days until her condition started to improve over the next two weeks. She was discharged after 17 days. At the outpatient consultation three months postoperatively, she had no particular complaints and was able to talk normally and walk without support and although not formally tested, there were no apparent language, motor, or cognitive deficits. An MRI brain scan showed (Figures 3(a)–3(d)) (i) no midline shift, marked enlargement of the left lateral ventricle and widening of the sulci on the left frontal, temporal, and parietal lobes and widened left sylvian fissure signifying presence of both subcortical and cortical brain atrophy; (ii) diffuse and focal periventricular white matter hyperintensities along the body and posterior horn of the lateral ventricle which may signify cerebral spinal fluid seepage or reperfusion infarcts; and (iii) left parietal bone signal changes which may be craniotomy-associated changes or bone infarction. There were no changes of the small cerebral infarcts on the right frontal lobe deep white matter compared to those observed in 2017. MRI angiography was normal with no features of arteriovenous malformations.

## 3. Discussion

Though silent cerebral infarctions are frequent, EDH in SCD is rare [2, 4, 8]. Based on data from ∼4000 SCD patients followed for an average of about 5 years, the Cooperative Study of Sickle Cell Disease found that those with HbSS were more frequently affected compared to other SCD genotypes and that the incidence of hemorrhagic stroke in HbSS patients was the highest among patients aged 20 to 29 years [8].

Here, we present a case of EDH in a SCD patient with previously reported silent cerebral infarction. Although there was no indication of trauma, sexual violence of young females is not uncommon in Tanzania [9]. A gynecological examination was therefore performed and found to be normal. Except for her underlying SCD, our patient had no other risk factors for bleeding or other comorbidities.

In contrast to previous reports of EDH in SCD, we had access to neuroimaging obtained prior to, during, and after the hematoma developed. We found that two years before the current event, small cerebral infarcts were evident. However, neither other forms of vasculopathy, including arteriovenous malformations, nor skull bone abnormalities were detected at that time. Notably, no vasculopathy was noted, either during her current hospital admission or three months after evacuation of the hematoma. The CT brain scan on admission revealed a subgaleal hematoma, possibly indicating a developing skull lesion/expansion due to increased hematopoietic activity leading to disruption of the skull cortex, which could be the cause of the EDH, supporting the notion suggested by Dahdaleh et al. [7]. We also noted the two hypodense lesions in the bone, suggesting that the bone had been affected.

Complications of the central nervous system rank as one of the most serious complications to SCD. Although EDH is rare, its long-term outcome can be debilitating [4, 5] and the associated mortality is high; in their review of 22 young SCD patients with EDH, Hettige et al. found that ∼1/3 of them died due to EDH [6]. Therefore, if EDH is suspected in a SCD patient, neuroimaging should be performed without delay, which is feasible also in low-resource settings such as ours [10].
